# Vaccination With Mouse Dendritic Cells Loaded With an IpaD-IpaB Fusion Provides Protection Against Shigellosis

**DOI:** 10.3389/fimmu.2019.00192

**Published:** 2019-02-08

**Authors:** Olivia Arizmendi, Prashant Kumar, Qi Zheng, Jason P. Stewart, William D. Picking, Wendy Picking, Francisco J. Martinez-Becerra

**Affiliations:** ^1^Higuchi Biosciences Center, University of Kansas, Lawrence, KS, United States; ^2^Department of Pharmaceutical Chemistry, University of Kansas, Lawrence, KS, United States

**Keywords:** *Shigella*, vaccine, fusion protein, dmLT, dendritic cell

## Abstract

Diarrheal diseases are a major cause of morbidity and mortality worldwide. They are most prevalent in settings with inadequate sanitation, poor hygiene and contaminated water. An important diarrheal pathogen in such settings is *Shigella*. No commercially available vaccine exists against shigellosis and immunity to the pathogen is serotype-restricted. We have previously shown that a polypeptide fusion of the Type Three Secretion Apparatus (T3SA) proteins IpaB and IpaD (named DBF) was efficacious as a vaccine against *Shigella*. Vaccination using different administration routes indicated that protection conferred by DBF did not fully correlate with antibodies. To define the immune responses involved in protection, we studied cellular responses to intranasal immunization with the DBF and the adjuvant dmLT. We found dendritic cell (DC) activation at the nasal associated lymphoid tissue (NALT). Activation markers CD86 and MHCII significantly increase in cells from immunized mice. Antigen exposure *in vitro* further confirmed the upregulation of CD80 and CD40 in primary dendritic cells. Animals immunized with antigen-primed dendritic cells were protected against *Shigella* infection, at levels comparable to the efficacy of immunization with the protein vaccine formulation. Therefore, we show that antigen-primed DCs are enough to provide immunity, and propose a mechanism of protection against *Shigella* spp. based on DC-mediated antigen presentation to T cells.

## Introduction

Shigellosis poses a significant public health burden, especially in developing countries. The most recent analysis of samples from Africa and Asia indicates *Shigella* spp. are a leading cause of diarrheal events in children under the age of 5 (1). Diarrheal disease caused by *Shigella* spp. and enterotoxigenic *Escherichia coli* (ETEC) account for more than 75,000 deaths in this age group each year (2). In older children and adults from the same region, *Shigella* spp. cause almost 90 million cases and 5 million hospitalizations each year (3). Improved sanitation and safe water supplies can reduce morbidity and mortality due to diarrhea; however, in impoverished settings these improvements are lacking due to poor infrastructure, high population density and low governmental involvement (4, 5). To overcome these challenges, the development of vaccines is necessary.

For *Shigella*, efforts to generate a commercially available vaccine are hindered by the presence of more than 50 serotypes of four different species (*S. flexneri, S. sonnei, S. dysenteriae, and S. boydii*). Subunit vaccines targeting conserved proteins can address this barrier by providing heterologous protection across multiple species/serotypes (6). Our group has developed a subunit vaccine based on the Type III Secretion System (T3SS) proteins of *Shigella* called IpaB and IpaD, which are well conserved across all species and serotypes. This subunit vaccine has been extensively tested in combination with the adjuvant dmLT, a double-mutant of the heat labile toxin of ETEC, as well as with other adjuvants (7–9).

The vaccine was further optimized with development of the chimeric protein DBF, which protects mice against pulmonary challenge with *S. flexneri, S. sonnei* and *S. dysenteriae* (10, 11). DBF is able to elicit comparable titers of protein-specific IgG and IgA antibodies to those of the combination formulation IpaB+IpaD. However, certain markers of Th1/Th17 polarization are further elevated in the spleen when DBF is used for immunization. These markers include the presence of IFN-γ secreting cells, increased secretion of IL-17A and decreased secretion of IL-4 in splenocytes in response to antigens *in vitro* (10). While protective efficacy against challenge with *S. flexneri* and *S. sonnei* were comparable between both versions of the vaccine, only DBF provided protection against *S. dysenteriae*, the toxin-producing *Shigella* spp. that causes severe dysentery and hemolytic uremic syndrome. Furthermore, a second study that compared different vehicle preparations with DBF+dmLT showed a better protective efficacy with Lauryldimethylamine N-oxide (LDAO) relative to the n-Octyl-oligo-oxyethylene (OPOE)-containing vehicle (11). Immunization with either elicited almost identical IgG titers but significantly higher splenocyte secretion of IL-17A was observed in the LDAO formulated protein, which highlights the potential role of cell mediated immunity for protection.

In this study, we further dissect the role of cellular immunity in the antigenicity and protective efficacy of DBF and its combined formulation with dmLT. Protective immunity conferred by dendritic cells (DCs), T-cells and B-cells is recognized as a hallmark of both resolution of natural infection and vaccination. In the case of *Shigella* spp. bacterium-specific cell mediated responses are primarily due to the generation of Th1/Th17 CD4+ cells (12, 13). Whereas, primary infection with *S. flexneri* induces differentiation of CD4+ cells to Th17 cells that produce IL-17A and IL-22, secondary infection also produces Th1 cells that secrete IFN-γ. CD4+ cell stimulation assays did not detect IL-4, denoting a lack of polarization toward Th2 lineage. Priming of Th17 cells was via MHCII and IL-6 cues by antigen presenting cells (13).

Immunization can also mimic these primary responses present during infection. For example, it has previously been shown that an attenuated *Shigella dysenteriae* strain used as a vaccine elicited Th1/Th17 responses (14). Macrophages from immunized animals secrete significantly higher amounts of IL-6, IL-23, IL-12p70, and IL-1β, which in the context of antigen-presenting cells would create a polarization environment of CD4+ cells toward the Th1/Th17 lineages. Indeed, CD4+ cells isolated from spleens of immunized animals secrete higher levels of the canonical Th1 cytokine IFN-γ and Th17 cytokine IL-17A relative to controls. Modulatory cytokine IL-10 was also elevated, whereas Th2 cytokine IL-4 had no significant change between groups (14).

Therefore, we analyzed the responses at the site of immunization by antigen-primed DCs and T cells, as well as the profiles prompted by their interaction in a simplified *in vitro* model. Adoptive transfer was also used as an immunization trial, in which DCs delivered intranasally were able to confer protection against pulmonary challenge. The immune response elicited by this vaccination included the generation of memory T cells with a distinctive lack of antibody responses against the *Shigella* antigens. Our findings support the hypothesis that cell-mediated immunity elicited by DCs plays a crucial role for protection against *Shigella* spp. conferred by the DBF+dmLT vaccine.

## Results

### Intranasal Immunization With DBF+dmLT Triggers Activation of Dendritic Cells

**Figure 1:**
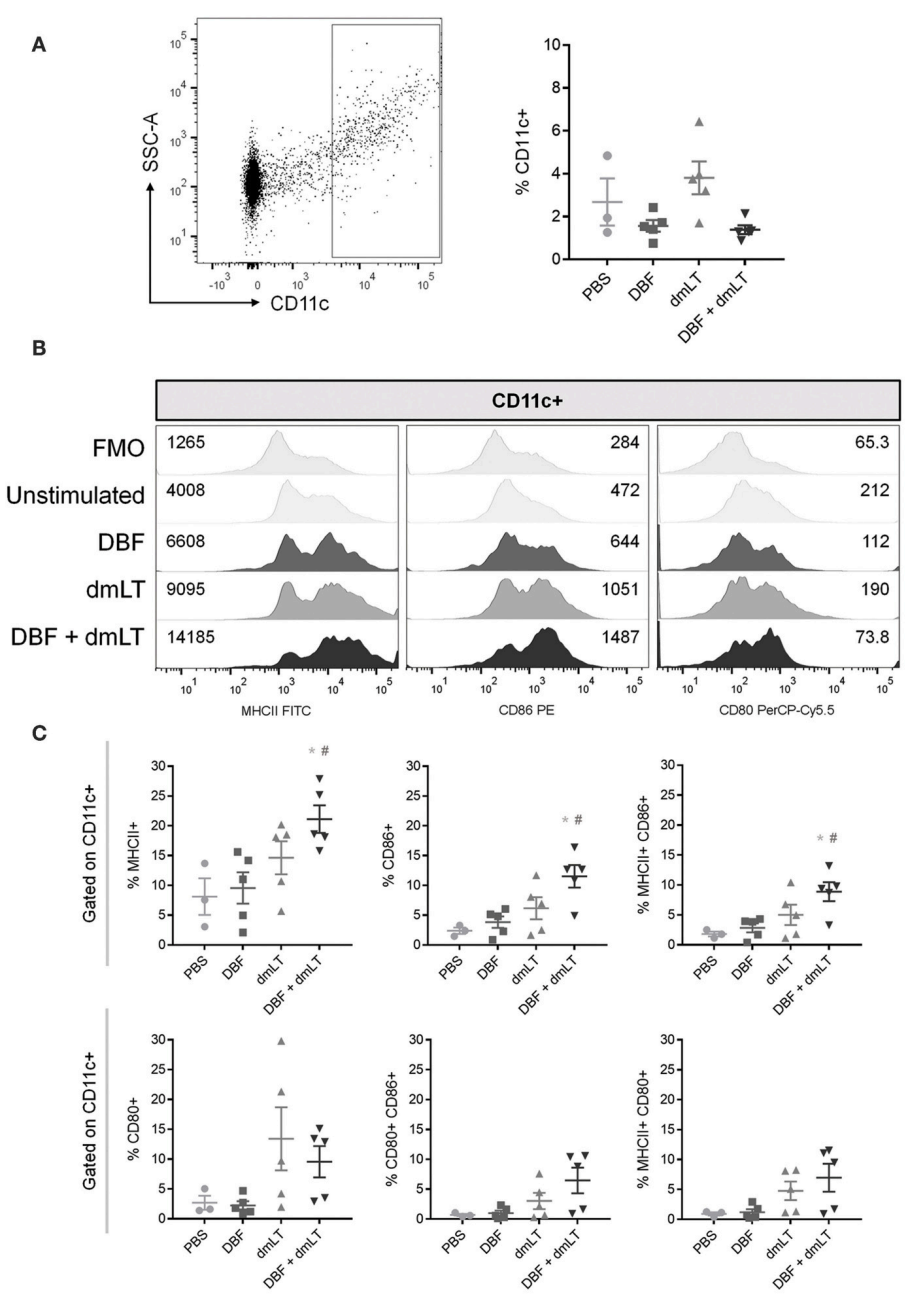
Dendritic cell populations in NALT post-immunization. Nasal-associated lymphoid tissue (NALT) was harvested from BALB/c mice 6 h after immunization with PBS, DBF, dmLT, and the combination DBF+dmLT. Cells in NALT were isolated and stained as described in Materials and Methods. **(A)** Dendritic cells were recognized by positive CD11c labeling (left). The percentage of CD11c+ cells was measured in NALT (right). **(B)** Expression of MHCII, CD86, and CD80 from a representative sample for each treatment. Fluorescence minus one (FMO) controls are shown for comparison. Numbers present inside the grids indicate median fluorescence intensity (MFI). **(C)** CD11c+ populations show differential percentages MHCII, CD86, and MHCII CD86 double positive cells. Each point represents a different individual. Error bars indicate standard deviation. Statistical significance was analyzed with an ordinary one-way ANOVA with a Tukey *post-hoc* test. All comparisons tested with an α value of 0.05. ^*^p < 0.05 vs. PBS, #*p* < 0.05 vs. DBF.

Mice were immunized intranasally with vaccine formulations of DBF either alone or adjuvanted with dmLT, or dmLT alone. A control group was administered PBS. After 6 h, the dendritic cell (DC) population found in the NALT was analyzed by flow cytometry (Figure 1). The percentage of CD11c+ cells remained unchanged across all groups (Figure 1A, right), however, their activation profile was altered as indicated by the levels of MHCII, CD86, and CD80. Immunization with DBF+dmLT produces significantly different activated DC populations to those elicited by DBF or dmLT alone. The median fluorescence intensity (MFI) values show an increase in MHCII and CD86 but not CD80, which showed a reduction in MFI in this group (Figure 1B). A marked increase is evident for levels of both MHCII and CD86 across treatments, with the highest levels observed for DBF+dmLT (Figure 1B, left and middle panels). These trends are not observed for CD80, as a reduction is observed in all groups (including DBF+dmLT) compared to PBS control (Figure 1B, right panel). Differential changes in levels of these activation markers point to the presence of different DC activation states depending upon the treatment. This is evidenced by a significant increase in the population positive for MHCII, CD86, or both in the group immunized with DBF+dmLT when compared to the PBS group (Figure 1C, top panels). An increase in the CD80+ and CD80/CD86+ populations in the group immunized with either dmLT or DBF+dmLT is apparent but not significant (Figure 1C, bottom panels). There are no significant increases for any of the measured markers in the groups immunized with the protein or adjuvant alone, suggesting that both components of the vaccine are needed to activate these cells.

NALT samples were also analyzed for differences in the number of T cells (Figure S1A) and macrophages (Figure S1B) and no significant differences were found across groups for either population. The activation state of each population was also measured as percent positive for CD69 in CD3+ cells or percent positive for MHCII in F4/80+ cells. No significant differences were found in the activation state of these populations across groups.

### Primary DC Stimulation Causes Activation Marker Upregulation

**Figure 2:**
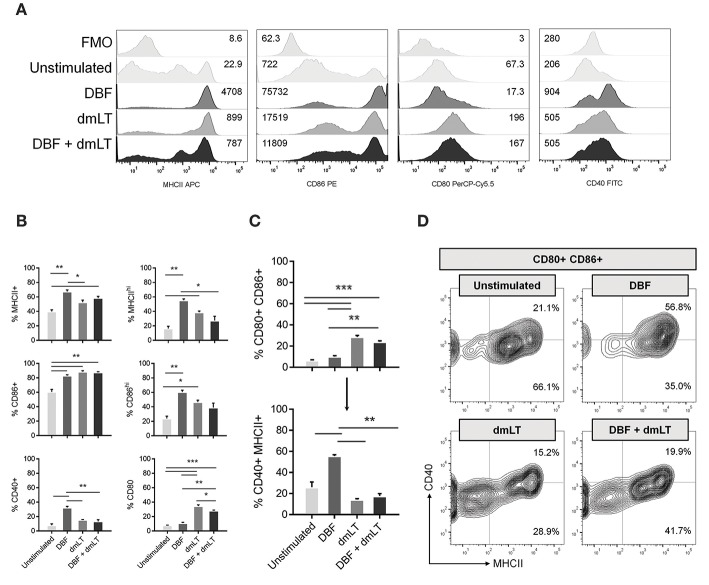
Antigen exposure promotes activation of bone-marrow derived DCs. Mouse bone marrow cells were differentiated into CD11c+ cells by 8-day culture in the presence of GM-CSF as described in Materials and Methods. **(A)** DC populations exposed to PBS, DBF, dmLT, and the combination DBF+dmLT were analyzed for expression of activation markers MHCII, CD86, CD80, and CD40 by flow cytometry. Stacked histograms show the fluorescence distribution and MFI values for each marker in a representative sample of each treatment. Some treatments induce a bi- or trimodal distribution of marker fluorescence. Fluorescence minus one (FMO) controls are shown for comparison. **(B)** Differences in positive populations for each marker were assessed by setting a cut-off of negative fluorescence with FMO samples. Percent positive populations for MHCII, CD86, CD40, and CD80 are shown using that criterion. Additionally, trimodal distributions for MCHII and CD86 allowed for an alternative histogram gate for distinct populations MHCII^hi^ and CD86^hi^. Their differences across treatments are also shown. **(C)** Percentages of the total DC population were analyzed for co-expression of CD80 and CD86 (top graph). The gated population double positive for CD80 and CD86 was further analyzed for co-expression of CD40 and MHCII (bottom graph). **(D)** Contour plots for CD80+ CD86+ populations further represent the differences in their distribution of MCHII+, CD40+, and MHCII+ CD40+ cells. Each bar represents the mean and SEM of duplicate biological samples. Statistical significance was analyzed with an ordinary one-way ANOVA with a Tukey *post-hoc* test. All comparisons tested with an α value of 0.05. ^*^p < 0.05, ^**^p < 0.01, ^***^p < 0.001.

To further define the activation state of DCs in response to these proteins, we measured the activation of primary cells in direct stimulation assays. Bone marrow derived DCs were stimulated *in vitro* for 48 h with DBF, dmLT, or the combination. A control group remained unstimulated by culture with PBS (Figure 2). Analysis of MFI values shows that treatment with DBF, dmLT, or the combination increase the levels of MHCII and CD86 in these cells (Figure 2A, left two panels). *In vitro*, the levels of CD80 increased in all groups that received dmLT with or without DBF (Figure 2A, third panel). The levels of CD40 incremented sharply in cells stimulated with DBF, while dmLT treated cells (alone or in combination) showed an intermediate increase (Figure 2A, right panel). When these cells are analyzed for the frequency of positive cells, significant increases in the MHC+ population were observed for cells stimulated with DBF and DBF+dmLT vs. control (Figure 2B, top left). When examined for CD86, all stimulated groups were significantly higher than the control (Figure 2B, center left). Interestingly, the histogram profiles showed a third population with distinctly higher levels of MHCII or CD86 for all samples. Stimulation with DBF led to higher frequencies of these MCHII^hi^ or CD86^hi^ populations, while stimulation with dmLT (alone or in combination) increased the intermediate population of these markers (Figure 2B, top and center right). CD40 is only significantly increased in cells stimulated with DBF, while the presence of dmLT drives surface expression of CD80 in these cells (Figure 2B, bottom left and right, respectively).

When the combination of markers is analyzed, we detect a significant increase in the CD80+ CD86+ population in cells stimulated dmLT or with DBF +dmLT, while cells stimulated with DBF alone do not have increased percentages of this double positive population (Figure 2C, top). Analysis of the levels of CD40 and MHCII in the population positive for CD80 and CD86 shows that treatment influences the generation of cell populations positive for all four analyzed markers. Interestingly, the majority of CD80+ CD86+ cells in the dmLT treated groups are not CD40+ MHCII+ (quadruple positive), while the smaller population of CD80+ CD86+ cells in the DBF treatment are (Figure 2C, bottom). This is indicated in the density plots for MHCII vs. CD40 in the CD80+ CD86+ population, where a distinct higher frequency of MHCII+ cells is observed in DBF treated cells irrespective of CD40, while cells treated with DBF+dmLT have lower frequencies of MHCII positive cells (91.8 vs. 61.6%, Figure 2D, upper right). Again, the presence of three distinct populations for MHCII is evident in the DBF+dmLT group, even if the population is gated on CD80/CD86 double positives (Figure 2D).

For all markers, it is evident that DBF and dmLT do not act additively in the upregulation of DC activation signals. This indicates a unique interplay between DBF and dmLT in their activation effects. Furthermore, responses to DBF+dmLT were less pronounced than those for DBF on all markers except CD86.

### Distinct CD86 Populations Show Differences in Other Activation Signals

**Figure 3:**
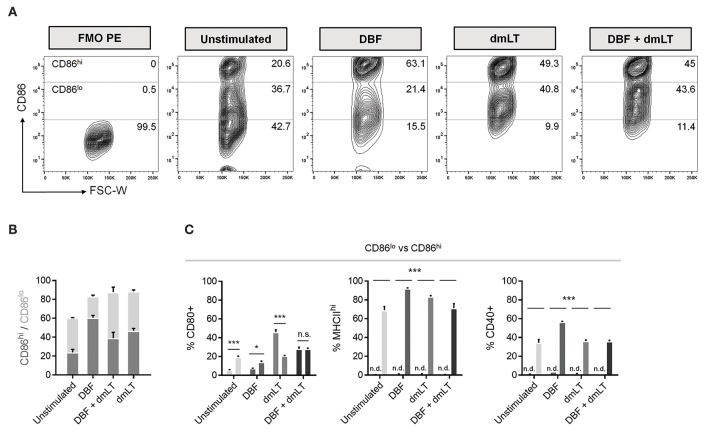
Differences in CD86^lo^ and CD86^hi^ DC populations. **(A)** DC populations exposed to PBS, DBF, dmLT and the combination DBF+dmLT show a trimodal distribution of CD86. Differences in the abundance of the CD86hi population are evident across treatments, with an enrichment in antigen-treated DCs. Numbers provided are percentages of total population. **(B)** Distribution analysis of the CD86+ DC population. **(C)** Comparison between CD86^lo^ and CD86^hi^ populations for activation markers CD80, MHCII^hi^ and CD40. CD80 is differentially distributed between the two populations, whereas all MHCII^hi^ and CD40+ cells were also CD86^hi^. Each bar represents the mean and SEM of duplicate biological samples. Statistical significance was analyzed with a 2-way ANOVA with a Sidak *post-hoc* test. All comparisons tested with an α value of 0.05. ^*^p < 0.05, ^**^p < 0.01, ^***^p < 0.001.

Population plots show marked distribution patterns of treated bone marrow-derived DCs with three CD86 surface levels: no increase, low (CD86lo) and high (CD86hi) surface expression (Figure 3A). These density plots provide further evidence that DBF provokes a radical shift toward elevated CD86 expression. A stacked graph illustrates that even though cells treated with all three protein combinations have comparable CD86+ percentages, high expression is more prevalent in the DBF treated sample (Figure 3B). Interestingly, when the other cell markers are analyzed based on CD86 distribution, CD80 is differentially distributed between CD86 levels depending on treatment, with dmLT again eliciting discreet cell surface marker upregulation with a significantly higher CD80+ population in those with moderate CD86 (CD86lo population) (Figure 3C). Treatment with DBF+dmLT yields the presence of CD80+ population evenly divided in CD86hi and CD86lo populations. For all samples treated with proteins, MCHII^hi^ and CD40+ populations are exclusively found in the CD86hi category.

### Specific Patterns of T Cell Polarizing Cytokines Are Induced by DC-Mediated Activation

**Figure 4:**
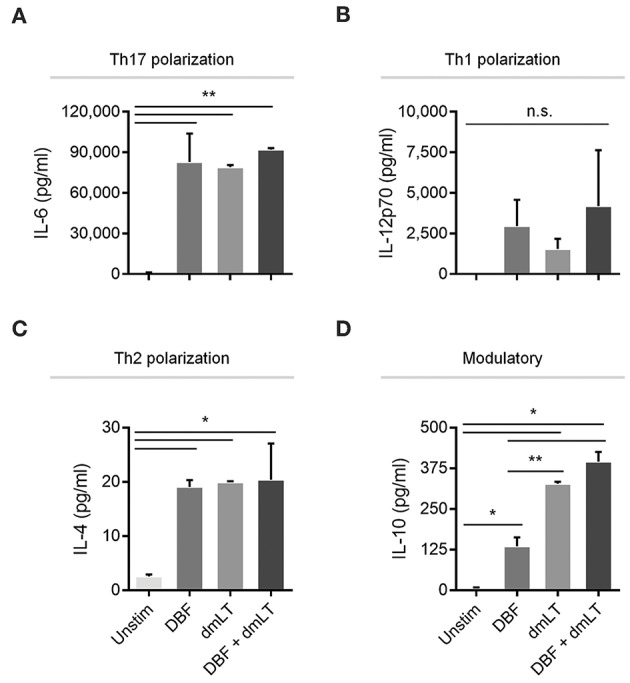
Cytokine secretion by DC after various treatments. DC populations exposed to PBS, DBF, dmLT, and the combination DBF+dmLT show different patterns of cytokine secretion and are presented according to their T-cell polarization role. A. IL-6, a cytokine critical for Th17 lineage commitment is enriched in all antigen-treated populations 2 h post-treatment. All samples are significantly different than PBS. **(B)** Th1-polarizing cytokine IL-12p70 is not significantly different across treatments. **(C)** Th2-polarizing cytokine IL-4 was significantly different in all antigen-treated samples vs. PBS. **(D)** IL-10 had an incremental secretion pattern, with all antigen-treated samples significantly different than the PBS control, and significant differences between DBF and dmLT or DBF+dmLT. Each bar represents the mean and SD of duplicate biological samples. Statistical significance was analyzed with an ordinary one-way ANOVA with a Tukey *post-hoc* test. All comparisons tested with an α value of 0.05. ^*^p < 0.05, ^**^p < 0.01.

To investigate whether the differences in the patterns of activation markers in dendritic cells could differentially influence polarization of Th cells, well-defined cytokines were measured in the supernatants of stimulated bone marrow-derived dendritic cell (BMDC) cultures (Figure 4). High expression levels of IL-6, a cytokine critical for Th17 polarization, was observed in samples stimulated with all proteins tested, while PBS controls showed negligible amounts of this cytokine (Figure 4A). Conversely, highly variable in levels of the Th1 polarization cytokine IL-12p70 were found across groups but no significant differences were found among treatments (Figure 4B). The Th2 polarization cytokine IL-4 was found only in stimulated samples, similar to the effect seen for IL-6, although at much lower levels (Figure 4C). In the case of modulatory responses, cumulative increases were measured for IL-10 for DBF, adjuvant and then the combination, with significant differences across all treatments (Figure 4D).

### Stimulation of DCs Causes Different Proliferation and Cytokine Patterns in T Helper Cells

**Figure 5:**
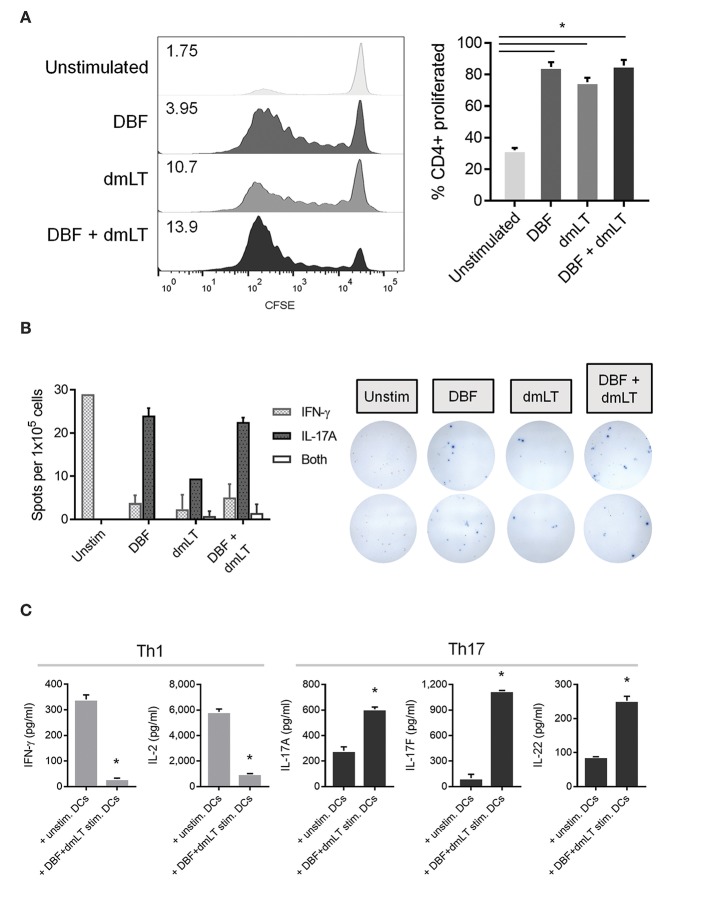
Co-culture of pre-treated DCs and isolated spleen T helper cells. DC populations exposed to PBS, DBF, dmLT and the combination DBF+dmLT for 48 h were then co-cultured for 7 days with CD4+ cells isolated from spleens of mice immunized with DBF+dmLT intranasally. **(A)** Proliferation of CD4+ cells was analyzed by CFSE fluorescence patterns. Stacked histograms show the differential distributions of fluorescence intensity. Numbers present inside the grids indicate the proliferation index of each population (proliferation index = average number of cell divisions during treatment). Bar graph shows the percentage of cells that proliferated after day 0. All antigen-treated DC co-cultures had significantly higher percentages of cell proliferation. Each bar represents the mean and SEM of duplicate biological samples. **(B)** CD4+ cells were sorted post-culture and cytokine-secreting cells determined by ELISpot. IFN-γ, IL-17, or double IFN-γ+IL-17 spot forming cells (SFC) were counted and bar graphs show the differential amounts present for each treatment. Representative well images are included, red spots are IFN-γ SFCs and blue spots are IL-17 SFCs. Each bar represents the mean and SD of duplicate biological samples. **(C)** Cytokine secretion by co-cultured cells was measured as described in methods. Each bar represents the mean and SD of duplicate biological samples. Statistical significance was analyzed with an ordinary one-way ANOVA with a Tukey *post-hoc* test. All comparisons tested with an α value of 0.05. ^*^p < 0.05.

To test for the actual polarization effect of stimulated DCs on T cells, splenocytes from mice previously immunized intranasally with DBF+dmLT were obtained and the CD4+ population isolated. These cells were subsequently incubated with BMDCs previously treated with DBF, dmLT, and the combination. These activated Th cells showed differential patterns of proliferation depending on the treatment of the DCs stimulating them (Figure 5A). All protein treated samples show several cell divisions indicative of active proliferation, whereas the PBS sample did not (Figure 5A, left). Although all treated DCs promoted a 7-day proliferation in more than 75% of CD4+ cells (Figure 5A, right), their proliferation indices show an additive effect for DBF and dmLT (Figure 5A, left); which is suggestive of an antigen dosage-independent effect.

The frequencies of CD4+ cells secreting Th1 cytokine IFN-γ and Th17 cytokine IL-17A were analyzed by ELISpot and compared across groups. CD4+ cells cultured with unstimulated DCs are exclusively IFN-γ secreting cells. Conversely, the frequency of cells distributes heavily toward IL-17A secreting cells when DBF, dmLT or the combination is used. We observed an overall reduction in the amount of secreting cells (irrespective of cytokine) in the dmLT only sample. Interestingly, a small population of dual-secreting cells was found in samples with dmLT with or without DBF (Figure 5B).

The amount of cytokines secreted in the supernatant by these co-cultures was then measured in a multiplex assay. The pattern of IFN-γ vs. IL-17A secretion observed in the ELISpot assays was confirmed in co-cultures of T cells with unstimulated vs. DBF+dmLT stimulated DCs, and levels are markedly different even when treatment groups are compared for each cytokine (Figure 5C). IFN-γ is mostly present in T cells cultured with unstimulated DCs. Similarly, IL-2 is significantly higher in samples with unstimulated DCs (Figure 5C, left panels) whereas samples with DBF+dmLT stimulated DCs, in addition to high secretion of IL-17A, had significantly higher levels of Th17 cytokines IL-17F and IL-22 (Figure 5C, right panels).

### DCs Stimulated With DBF +dmLT Protect Mice in a *S. flexneri* Challenge

**Figure 6:**
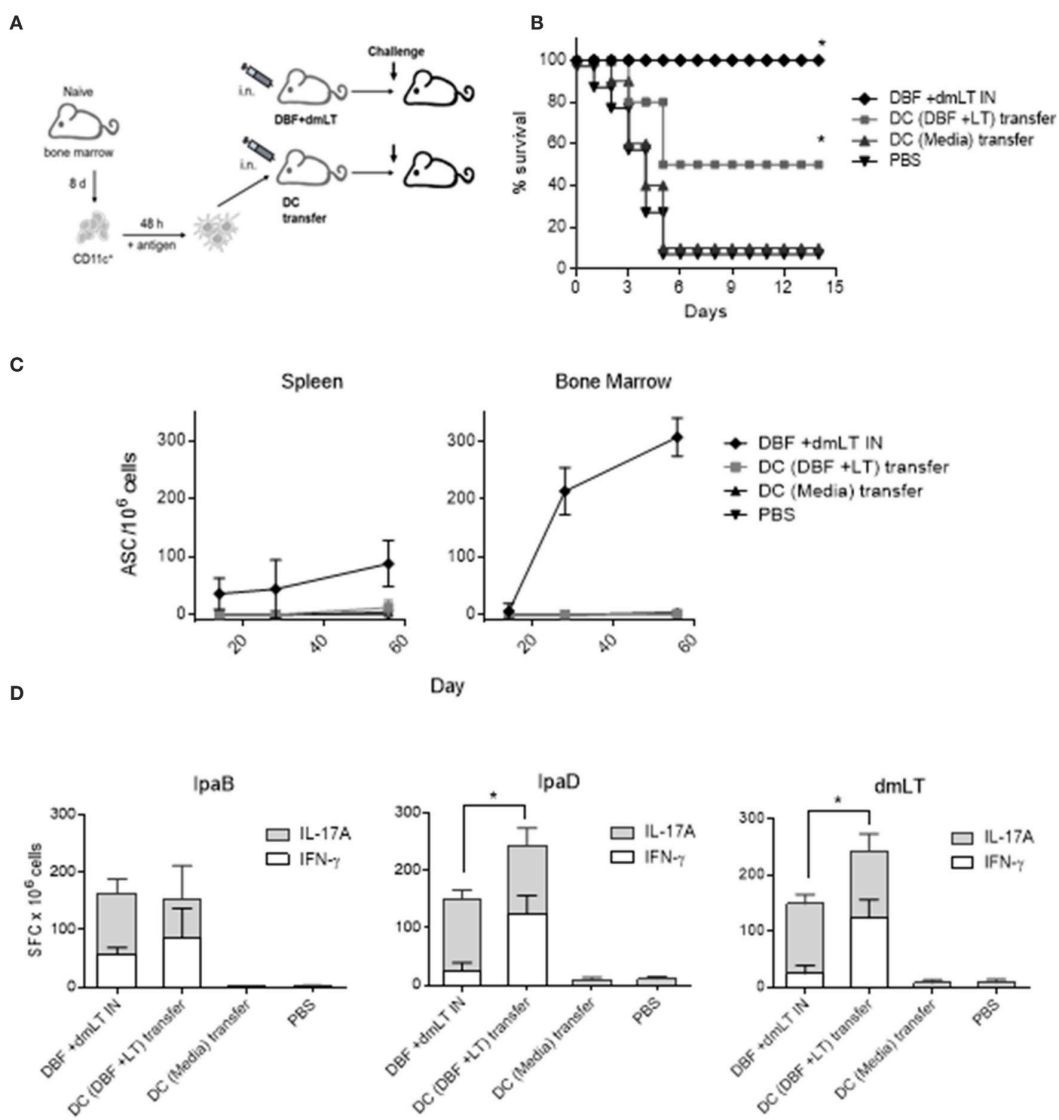
Adoptive transfer of DCs. **(A)** Immunization layout employed for transfer experiments. **(B)** Dendritic cells stimulated with PBS, or the combination DBF+dmLT for 48 h were administered intranasally to mice. Mice (*N* = 10) were challenged with 2 × 10^7^ CFU of *Shigella flexneri* 2457T. Animals immunized three times intranasally with DBF+dmLT or PBS were used as positive and negative controls, respectively. Survival was monitored for 14 days. ^**^p < 0.01 compared to survival of mice vaccinated with PBS using log-rank Mantel-Cox test, n.s. not significant. **(C)** Splenocytes and bone marrow cells from mice that were transferred DCs or vaccinated with protein were used in an ELISpot measuring IgG secreting cells specific for the DBF chimeric portion IpaB. **(D)** Splenocytes were stimulated with IpaB, IpaD or dmLT for 48 h. cytokine-secreting cells were determined by ELISpot. IFN-γ and IL-17A spot forming cells (SFC) were counted and bar graphs show the differential amounts present for each treatment. Each bar represents the mean and SD of biological replicates (five individuals/group). Statistical significance was analyzed with a 2-way ANOVA with a Sidak *post-hoc* test. All comparisons tested with an α value of 0.05. ^*^p < 0.05 in the frequencies of IFN-γ secreting cells.

We determined the potential of DCs to generate protective responses by intranasal adoptive transfer of DCs unstimulated or stimulated with DBF+dmLT. We decided not to transfer cells treated with DBF without adjuvant as we previously demonstrated that DBF by itself is not able to provide protection in mice (10). These cells were transferred three times 14 days apart to 15 mice. Additionally, various vaccination groups were probed for protective efficacy in a lethal pulmonary challenge with wild-type *S. flexneri* (Figure 6A) after 56 days of the first immunization. After the challenge, the control group receiving DBF+dmLT formulation intranasally showed 100% protection. As expected, most of the PBS immunized mice succumbed to *S. flexneri* challenge, showing only 10% survival. While the unstimulated DCs did not provide any protection against *S. flexneri*, the DCs stimulated with DBF +dmLT provided a 50% protection against challenge (Figure 6B). This difference was significantly different from the DC media transferred group and PBS negative controls. We analyzed the immune responses in these mice in order to characterize the responses generated by the antigen stimulated dendritic cells. We analyzed the frequencies of antibody secreting cells (ASCs) specific for the delivered antigens in the spleen and bone marrow of immunized mice via ELISpot assays (Figure 6C). High frequencies of IgG secreting cells were detected in mice that received the DBF and dmLT intranasally. In contrast, the group that received DCs incubated with DBF+dmLT did not show any detectable ASCs in these organs. Mice that received unstimulated DCs or PBS intranasally did not show detectable ASCs specific for IpaB. The lack of ASCs in the DC transferred mice was confirmed by measuring IgG in serum using ELISA (not shown). In order to characterize the T cell responses in these mice we performed dual color ELISpots to measure IFN-γ and IL-17A secreting cells specific for IpaB, IpaD and dmLT. Splenocytes obtained from mice that received unstimulated DCs showed no antigen specific cytokine-secreting cells. Frequencies for IFN-γ- and IL-17A-secreting cells specific for IpaB are similar in splenocytes from mice that received proteins intranasally or that received DCs incubated with DBF +dmLT (Figure 6D). The same samples stimulated with IpaD or dmLT have significantly more IFN- γ secreting cells in the mice that received DCs incubated with DBF +dmLT. The frequencies in IL-17A secreting cells specific for IpaD or dmLT were similar. The negative controls in these groups did not show significant frequencies of cytokine secreting cells.

### Lack of T Cells in Vaccinated Mice Reduces Protective Efficacy Against a *Shigella* spp. Challenge

**Figure 7:**
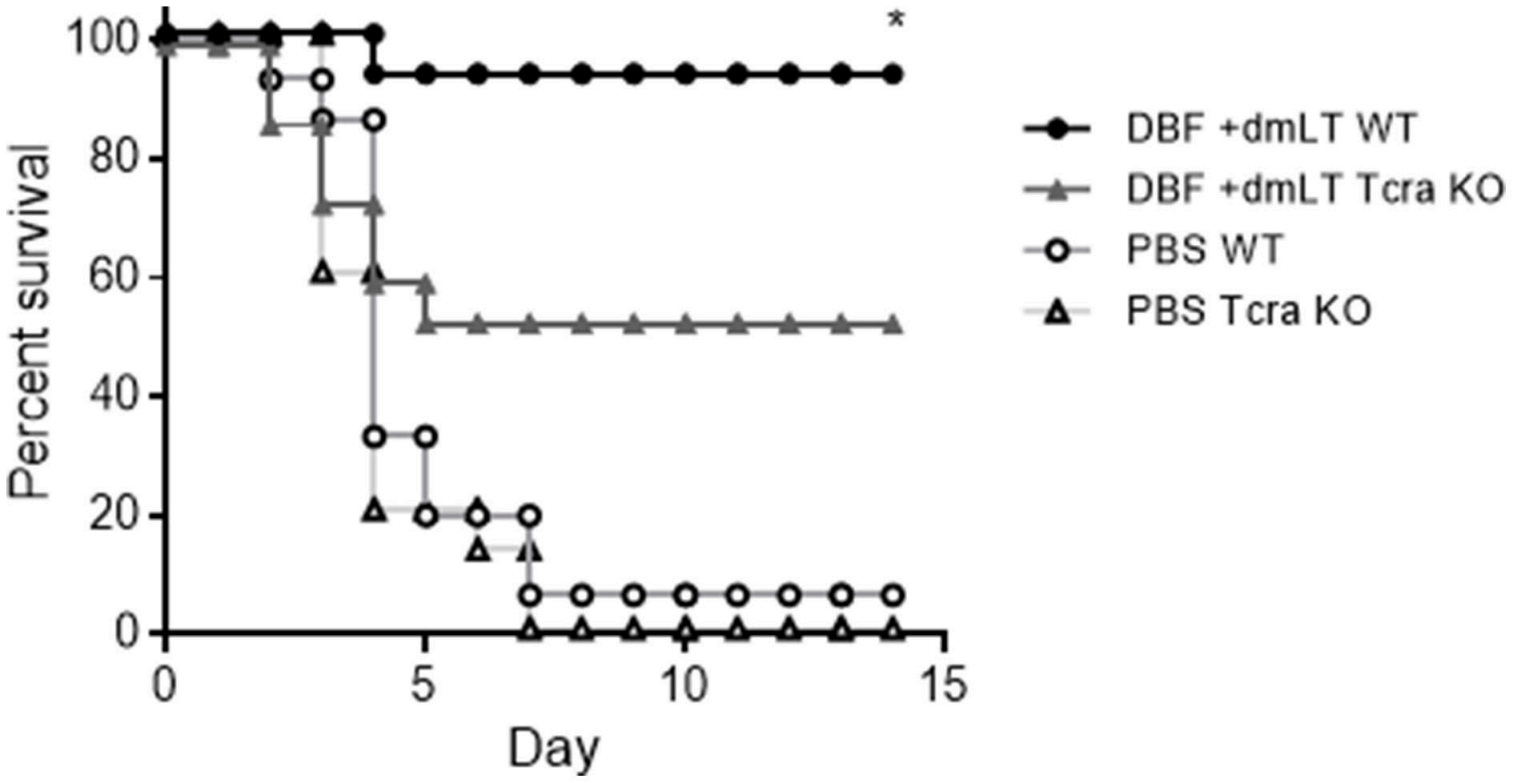
Immunization of T cell deficient mice. Mice (*n* = 15) were immunized three times at days 0, 14, and 28 before challenge with PBS or the combination DBF+dmLT intranasally,. Mice were challenged with 2 × 10^7^ CFU of *Shigella flexneri* 2457T. Survival was monitored for 14 days. ^**^p < 0.01 compared to survival of KO mice vaccinated with DBF +dmLT using log-rank Mantel-Cox test.

To further explore the role of cellular immunity in protection conferred by DBF protein, we performed vaccinations on mice lacking T cells (Tcra^tm1Mom^) along with their control in C57BL-6 background (WT). These mice were immunized three times with either DBF +dmLT or PBS intranasally. At day 56, mice were challenged with *S. flexneri* and survival was measured. Wild type and mutant mice immunized with PBS did not exhibit any differences in survival when challenged with *S. flexneri*. The wild type mice that received DBF +dmLT showed a 90% protection, while the Tcra mice had a reduction of 50% in their survival (Figure 7).

## Discussion

The magnitude of the morbidity and mortality of shigellosis in developing countries underscores the need for a safe, effective and inexpensive vaccine. The reason for the lack of a commercially available bradly protective *Shigella* vaccine is multifactorial, contributed to by a poor understanding of the correlates of protective immunity (15). Some studies exist exploring the surrogates of protective immunity against attenuated *Shigella* strains, but the majority of these responses are representative of serotype-restricted immunity. Most are primarily driven by O-antigen in the form of antibodies (6); however, a live hybrid *Shigella* vaccine was found to elicit elevated splenocyte secretion of Th1 and Th17 cytokines. Little information exists about the surrogates of protection of subunit vaccines against shigellosis and only descriptive data of cellular immunity responses are available. For example, our previous studies on DBF show an increase in IFN-γ and IL-17 secreting cells in the spleen (10, 11), whereas IFN-γ was also found in lung lavages of mice immunized with another subunit vaccine based on purified OmpA (16, 17). Therefore, our study had the goal of describing the immune responses sufficient for protection provided by the subunit vaccine DBF when adjuvanted with dmLT.

We have previously reported the efficacy of intranasal immunization with a DBF+dmLT formulation against various *Shigella* spp. challenges, thus, this route was used as our experimental standard. Upon intranasal immunization, cell populations in the NALT are responsible for antigen uptake, cytokine secretion and transport to draining lymph nodes (dLN) (18). Furthermore, this route has been shown to induce Th17 stimulation, independent of adjuvant choice (19). Indeed, mice immunized intranasally with DBF+dmLT contain antigen presenting cell populations in the NALT that are discernibly different from those of unimmunized animals. CD11c+ cells, although found in similar numbers across treatments, showed increased activation profiles in DBF+dmLT immunized animals, as exhibited by elevated MHCII and CD86 surface expression. Remarkably, both DBF and dmLT are needed for these activation profiles. Although CD3+ and F4/80+ populations are not enriched in the NALT of immunized animals, their activation profiles in individual mice show interesting features. Percentages of CD3+ CD69+ and F4/80+ MHCII+ cells remain homogeneous among individuals immunized with DBF or those unimmunized but become heterogeneous upon immunization with dmLT or DBF+dmLT. This could be due to the timing of responses present in the NALT, as migration of macrophages to the dLN might still be en route 6 h post-immunization. The activation feature of the DBF protein represents an interesting characteristic of this protein. This activity is not related to LPS contamination as our purification scheme includes anion exchange columns and use of a detergent for stabilization. With these procedures, the total levels of endotoxin are consistently under 0.1 E.U. More probably, this activity can be related to the intrinsic activity of IpaB and IpaD, which are able to activate macrophages (20, 21).

DC response to antigen treatment was elevated when a simplified *in vitro* model was used. Antigen-treated CD11c+ populations are, as in the NALT, positive to MHCII and CD86. However, the appearance of a third population with higher expression of these markers (i.e., trimodal distributions) indicate the population is being drawn toward a highly activated state. Furthermore, CD80 is now differentially expressed by dmLT and DBF+dmLT treated DCs and this is preferentially seen for dmLT in populations with a moderate level of CD86 (CD86lo). Kinetics of CD80 and CD86 expression in DCs have been shown to influence the phenotype of their primed T cells, primarily in markers of antigen responsiveness. A CD86 response preferentially induces sustained T cell activation and a CD80 response promotes inhibitory signaling due to its increased affinity to CTLA-4 (22). Thus, the occurrence of elevated CD80 responses in dmLT-treated samples in our *in vitro* system might be counterproductive to T cell stimulation. The differences between our *in vivo* and *in vitro* experiments could be related to a difference in concentration reached in tissues vs. the ones used in culture. Another difference is the presence of distinct DC populations in resident tissue that respond to these proteins, whereas a single homogeneous DC population is used for our *in vitro* experiments.

In vaccine studies, the stimulation of Th populations that respond in a manner similar to a natural infection is desirable. Previous studies with transgenic mice have shown that IFN-γ-secreting T and NK cells are necessary for bacterial clearance during a *Shigella* infection, and ultimately, for survival (23, 24). Newer evidence points to the role of IL-17A in reducing the bacterial load upon reinfection, which induces a 6:1 balance of Th17 and Th1 populations at the site (13). Our results show antigen treatment of DCs induces a primarily Th17-promoting environment via increased IL-6, as confirmed by CD4+ populations that secrete IL-17A, IL-17F, and IL-22. Also, DC treatment could restrict Th1 differentiation and T cell activation due to the effects of IL-10 (25). In contrast, untreated DCs showed *in vitro* a Th1 polarization bias, evidenced by the marked reduction in Th17 responses.

Our challenge data supports the idea that activated DCs, obtained by culture with the antigens present in our vaccine formulation DBF and dmLT are able to protect against *Shigella* infection albeit at a lower extent than intranasal immunization does. T cells that are generated *in vivo* after transfer of antigen-primed DCs mainly show secretion of IFN- γ or IL-17A in levels comparable to the protein based immunization. The difference in protection suggests that other responses are required for the levels of protection observed with the proteins. The lack of humoral responses to IpaB and IpaD would suggest that both cellular and humoral responses are required in tandem to generate the most efficient protection in this vaccine. The site of DC transfer also plays a role in the outcome of the vaccinations, as experiments where DCs were transferred via intraperitoneal injection failed to provide protection (data not shown).

These conclusions are supported by the incomplete reduction of protection observed in the Tcra KO mice. Humoral responses in these mice could provide partial protection in the absence of T cell mediated responses. It is relevant then to probe the mechanism of antibody-mediated protection in this vaccine.

In conclusion, the DBF protein is able to activate DCs in conjunction with the adjuvant dmLT in a way that promotes polarization of T cells to a Th1 or Th17 profile. We also provide evidence that intranasal transfer of activated DCs generate DBF specific T cells that allow mice to survive a lethal *Shigella* challenge (50% survival rate), with a reduced efficacy probably due to a lack of humoral responses. Therefore, the protective effect of our vaccine is partially achieved by cell-mediated immunity.

## Materials and Methods

### Materials

Antibodies were from Tonbo Biosciences, Biolegend or BD Biosciences. 5-(and−6)-Carboxyfluorescein diacetate succinimidyl ester (CFSE) was from Tonbo Biosciences (San Diego, CA). EasySep PE selection kit was from STEMCELL (Vancouver, Canada). Purification columns HisTrap Crude FF and HiTrap Q FF were from GE Healthcare (Piscataway, NJ). U-PLEX kits were from Meso Scale Discovery (Rockville, MD). Double-color ELISPOT kits were from Cellular Technology Limited (Cleveland, OH). N,N-Dimethyldodecylamine N-oxide (LDAO) solution was from Sigma-Aldrich (St. Louis, MO). Limulus Amebocyte Lysate (LAL) endpoint chromogenic kit was from Lonza (Allendale, NJ). All cell culture plastics were TPP brand from MidSci. Recombinant premium-grade GM-CSF was from Miltenyi Biotec (San Diego, CA). RPMI-1640 and fetal bovine serum were from Mediatech (Manassas, VA).

### Protein Purification

Recombinant protein expression was carried out as previously described (10). Briefly, *E. coli* Tuner DE3 cells were transformed with DBF/pET28a, grown in culture, and expression induced. The purification scheme included IMAC and anion-exchange chromatography. Protein was tested for presence of LPS by Limulus Amebocyte Lysate (LAL) assay and determined to be LPS free (< 0.1 EU/ml). All proteins were stored at −80°C until use. Adjuvant dmLT was provided by John Clements at Tulane University.

### Animal Use Statement

Animals were housed and handled in agreement with University of Kansas' Institutional Animal Care and Use Committee practices (protocol AUS 221-01).

### Mice IN Immunizations

Female BALB/c Mice (6–8 weeks old) or C57BL/6J and B6.129S2-Tcratm1Mom/J (Tcra) from Jackson Laboratory were anesthetized using isoflurane delivered via a vaporizer and immunized intranasally (20 μl volume) three times in 2-week intervals with 20 μg of DBF with 2.5 μg of dmLT in sterile PBS.

### Challenge

Mice (10/15 per group) were anesthetized with isoflurane and then challenged intranasally with 2 × 10^7^ CFU of *S. flexneri* in 20 μl. Animals were monitored for weight loss and health score for 14 days. Mice that lost more than 20% of their starting weight for more than 48 h or remained too sick were humanely euthanized. These experiments were performed twice with similar results.

### Cell Staining and Flow Cytometry

Antibodies used were anti-CD80 PerCP-Cy5.5 Clone 16-10A1 and anti-CD86 PE Clone GL-1 from BD Biosciences; anti-CD40 FITC Clone 3/23 from Biolegend; and anti-CD11c APC-Cy7 Clone N418, anti-MHCII APC Clone M5/114.15.2, anti-F4/80 PE Clone BM8.1, anti-CD69 FITC Clone H1.2F3, anti-CD3 VioletFluor450 Clone 17A2, anti-MHCII FITC Clone M5/114.15.2, anti-CD4 PE Clone GK1.5, and anti-CD16/CD32 Clone 2.4G2 (Fc-block) from Tonbo Biosciences. Isolated cells were stained following a basic protocol based on a method previously described (26). Briefly, cells were washed with 2% BSA in PBS and incubated with Fc-block reagent for 10 min on ice. Then, cells were incubated with appropriate antibodies for 30 min and washed twice with 2% BSA in PBS. All cell suspensions were analyzed with a BD FACSAria Fusion. Fluorescence minus one (FMO) samples were used to set the gates for each marker. Data analysis of flow cytometry data was performed with FlowJo (Ashland, OR) or FCS Express (Glendale, CA) software.

### Dendritic Cell Isolation

Femurs of female BALB/c mice were used for bone marrow isolation and differentiation into CD11c+ cells based on a method previously described (27). Briefly, bone marrow cells washed with cold media were treated with red cell lysis buffer and strained with a nylon mesh. A single cell suspension was grown in the presence of GM-CSF (20 ng/ml) for 8 days. On day 3, floating cells were gently removed. On day 8, adherent macrophages and dendritic cell in suspension were evident; only suspension cells were recovered. These cells were determined to be immature dendritic cells by staining with an anti-CD11c antibody. Cell populations were consistently >95% CD11c positive.

### Dendritic Cell Stimulation

1 × 10^6^ immature dendritic cells were seeded onto 60 mm tissue culture dishes in RPMI-1640 medium containing 10% FBS and penicillin/streptomycin and stimulated as described before (26). Briefly, cells were incubated for 48 h with recombinant proteins at a concentration of 10 μg/ml. After stimulation, cells were harvested and stained for cell surface markers. Cell supernatant was collected and frozen at −20°C for cytokine secretion analysis.

### T Cell Co-culture

CD4+ memory T cells were isolated from spleens of BALB/c mice previously immunized intranasally with DBF+dmLT (day 56 after first immunization) as previously described (14, 26, 28). A single cell suspension was stained with a PE anti-mouse CD4 antibody as described above. Then, cells were incubated in a solution 2% FBS 1 mM EDTA in PBS with a PE selection cocktail. CD4+ cells were isolated by positive selection with magnetic particles, washed and resuspended in PBS. These cells were then stained with a 5-(and−6)-Carboxyfluorescein diacetate succinimidyl ester (CFSE) solution for proliferation studies. 1 × 10^6^ stimulated dendritic cells and 5 × 10^5^ T helper cells were then co-incubated in a 24-well plate with media for 7 days. CD4+ cells were analyzed for CFSE labeling after co-culture and sorted for ELISpot assays with a BD FACSAria Fusion.

### Dendritic Cell Immunizations

Mice were anestethized using isoflurane. Cultured dendritic cells were administered intranasally at days 0, 14, and 28 of the study (based on 24, 26). Briefly, antigen-stimulated dendritic cells were harvested and washed three times with sterile PBS. 1 × 10^6^ cells in 30 μl of PBS were applied to nares of anesthetized mice.

### NALT Isolation

Mice were euthanized and NALT tissue was extracted from the upper mouth cavity from individual mice (*N* = 5 per treatment/time point) under aseptic conditions and stored in complete RPMI. A single cell suspension was obtained by homogenization through a nylon mesh and washed with cold media. Staining for surface markers was done as described above.

### Cytokine Determinations

Supernatants collected from dendritic cell stimulations or co-cultures were analyzed with U-PLEX kits for cytokines IL-4, IL-6, IL-10, and IL-12p70; and IFN-γ, IL-2, IL-17A, IL-17F, and IL-22, respectively. Cytokine concentrations were determined with a MSD plate reader and associated software (Meso Scale Discovery).

### ELISpot

Sorted CD4+ cells from co-cultures were plated onto ELISpot plates coated with antibodies against IFN-γ and IL-17A for a dual-color assay. Cytokine-secreting cells were analyzed with a CTL immunospot reader (Cellular Technology Limited). Splenocytes from mice transferred dendritic cells were collected and restimulated with IpaB, IpaD, or dmLT for 48 h. These cells were then plated in ELISpot dual-color assay plates and assayed as described.

### Statistical Analysis

All graphs and statistical comparisons were done with GraphPad Prism (version 6 or 7). The data is represented as mean with SD or mean with SEM (when individual values were plotted). Representative data from one experiment is presented, *in vitro* experiments were performed at least 3 times and *in vivo* experiments performed twice. Appropriate tests were used for statistical analysis, including one-way and two-way ANOVA, and log-rank Mantel-Cox test. A *p* < 0.05 was considered significant in all comparisons.

## Ethics Statement

This study was carried out in accordance with the recommendations of University of Kansas' Institutional Animal Care and Use Committee practices. The protocol was approved by the University of Kansas IACUC (protocol AUS 221-01).

## Author Contributions

OA and FM-B designed experiments. OA, JS, QZ, PK, and FM-B performed experiments and acquired data and all authors drafted or revised the manuscript and have approved the final version. WiP and WeP provided overall guidance and consulted on experimental design and interpretation.

### Conflict of Interest Statement

The authors declare that the research was conducted in the absence of any commercial or financial relationships that could be construed as a potential conflict of interest.
