# Supplementary figures and images for: Vaccination With Mouse Dendritic Cells Loaded With an IpaD-IpaB Fusion Provides Protection Against Shigellosis
Source: Front Immunol. 2019 Feb 8;10:192. doi: 10.3389/fimmu.2019.00192 (PMC6376248; doi:10.3389/fimmu.2019.00192)

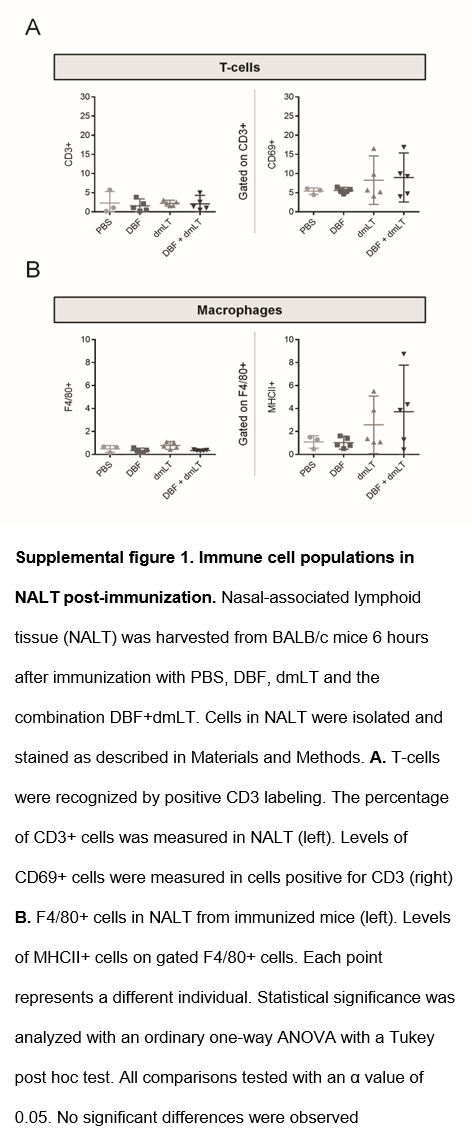

Supplement: Supplementary file 1 [file Image_1.tif]
